# The representation of material categories in the brain

**DOI:** 10.3389/fpsyg.2014.00146

**Published:** 2014-03-12

**Authors:** Richard H. A. H. Jacobs, Elisabeth Baumgartner, Karl R. Gegenfurtner

**Affiliations:** Allgemeine Psychologie, Justus-Liebig-UniversitaetGiessen, Germany

**Keywords:** material perception, categorization, fMRI, texture perception, MVPA, adaptation, physiological

## Abstract

Using textures mapped onto virtual nonsense objects, it has recently been shown that early visual cortex plays an important role in processing material properties. Here, we examined brain activation to photographs of materials, consisting of wood, stone, metal and fabric surfaces. These photographs were close-ups in the sense that the materials filled the image. In the first experiment, observers categorized the material in each image (i.e., wood, stone, metal, or fabric), while in an fMRI-scanner. We predicted the assigned material category using the obtained voxel patterns using a linear classifier. Region-of-interest and whole-brain analyses demonstrated material coding in the early visual regions, with lower accuracies for more anterior regions. There was little evidence for material coding in other brain regions. In the second experiment, we used an adaptation paradigm to reveal additional brain areas involved in the perception of material categories. Participants viewed images of wood, stone, metal, and fabric, presented in blocks with images of either different material categories (no adaptation) or images of different samples from the same material category (material adaptation). To measure baseline activation, blocks with the same material sample were presented (baseline adaptation). Material adaptation effects were found mainly in the parahippocampal gyrus, in agreement with fMRI-studies of texture perception. Our findings suggest that the parahippocampal gyrus, early visual cortex, and possibly the supramarginal gyrus are involved in the perception of material categories, but in different ways. The different outcomes from the two studies are likely due to inherent differences between the two paradigms. A third experiment suggested, based on anatomical overlap between activations, that spatial frequency information is important for within-category material discrimination.

## Introduction

Perception research has focused primarily on the perception of lines and shapes, and on the processing of complex objects such as faces. The largely neglected counterpart—the perception of materials and their properties—has come into focus only in recent years, but promises to yield important insights about basic visual and tactile perceptual processes.

An understanding of the perception of material properties is important for both theoretical and practical reasons. For instance, consider avoiding a slippery path. In addition, understanding what makes an object look wooden, can help in manufacturing products that are intended to look wooden. Theoretically, an understanding of material perception is important because it may be driven by mechanisms other than those involved in the processing of edges. Texture provides important cues to material composition, although other factors, such as reflectance and transparency also play a role. Therefore, we expect that earlier findings from texture perception research will generalize to material perception. An indication that material perception is different from shape or outline perception is provided by studies showing that certain brain areas are specialized in processing texture information and gloss (see below). Behavioral results also indicate separate channels for form and texture processing (Cant et al., 2008). Textures can be summarized withsummary statistics. Algorithms for extracting such statistics, and using them for generating perceptually identical textures (Balas, 2006) are available (Portilla and Simoncelli, 2000).

The research on material properties has found effects of texture (Julesz, 1981; Bhushan et al., 1997; Balas, 2006; Freyberger and Farber, 2006; Bergmann Tiest and Kappers, 2007; Lesch et al., 2008; Buckingham et al., 2009; Jacobs et al., 2012), and light reflectance properties (Boyaci et al., 2004; Ho et al., 2006; Motoyoshi et al., 2007; Anderson and Kim, 2009; Doerschner et al., 2010; Kim and Anderson, 2010; Fleming et al., 2011; Kim et al., 2011; Marlow et al., 2011) of surfaces on perception and action, mainly in the visual domain, but also in the tactile domain. Recently, the neuro-anatomical basis of material perception has been investigated. Hiramatsu et al. (2011) found that the pattern of voxel activations in the visual cortex can be used to successfully tell which materials an observer is viewing. Posterior visual regions (V1) were more informative than more anterior visual regions for predicting material categories, in accordance with the retinotopic layout of early visual regions. Much of the visual information entering the eye is propagated to the visual cortex, necessitating that material information be present at this stage to support material categorization. However, one would also expect higher order areas to be involved in categorizing materials, using neurons that code the material categories or the associated material properties, rather than just the retinotopic information. Moreover, there may be higher-level brain areas involved in categorization not only of materials but of things in general. Converging evidence from studies investigating semantic categorization, semantic fluency, semantic dementia, etc., points to the frontal and temporal lobes as areas that are involved in high-level categorization (Crowe, 1992; Frith et al., 1995; Curtis et al., 1998; Troyer et al., 1998; Hugdahl et al., 1999; Pihlajamäki et al., 2001; Grossman et al., 2002; Henry and Crawford, 2004; Baldo et al., 2006; Costafreda et al., 2006). Such areas could not turn up in Hiramatsu et al. (2011)'s study as they only scanned posterior, visual brain areas.

Other brain imaging studies have investigated aspects related to material perception, such as texture perception (Puce et al., 1996; Peuskens et al., 2004; Cant and Goodale, 2007; Cant et al., 2009; Cavina-Pratesi et al., 2010a,b), and roughness (Kitada et al., 2005) and gloss (Okazawa et al., 2012) perception. Evidence suggests that tactile roughness perception is primarily processed in the parietal operculum and insula (Kitada et al., 2005). Visual gloss appears to be processed in inferior temporal cortex, at least in macaques (Nishio et al., 2012; Okazawa et al., 2012). The brain imaging studies that investigated texture perception and those that studied patients with defective texture perception (Cavina-Pratesi et al., 2010a,b) suggest that brain areas around the collateral sulcus are involved in the processing of visual textures. The studies point to one posterior site in the visual cortex (Cavina-Pratesi et al., 2010a,b), and another more anterior site in the parahippocampal place area (Cant et al., 2009; Haak et al., 2010; Cant and Goodale, 2011). Single-cell recording studies in monkeys provide evidence for texture coding in area V4 and inferior temporal cortex (Wang et al., 2003; Arcizet et al., 2008; Köteles et al., 2008). Whether these brain areas contribute to texture perception, to material perception, or some related processes is unknown. Arnott et al. (2008) argued that the parahippocampal place area should be recognized as a material processing region, based on their finding that it responded to material-specific sounds, in addition to its responsiveness to visual textures. The findings of Hiramatsu et al. (2011), mentioned above, do not support a specific role for the parahippocampal area in the encoding of material categories or the associated material properties, although they provide evidence that the parahippocampal area, along with other relatively anterior regions, is involved in more perceptual aspects (i.e., judgments) of material processing. Together, the available findings point to the parahippocampal gyrus and visual cortex as places that may be involved in the perception of material categories, but areas in temporal (texture, gloss, categorization) and frontal cortex (categorization) may also play a role.

In the present paper, we aimed to elucidate brain regions involved in distinguishing between different materials. We expect the occipital cortex and the parahippocampal gyrus to be involved in the visual perception of materials and explore the rest of the brain for additional regions sensitive to materials. Since we expect neurons that code for different materials to be spatially intermingled, regular contrasts between material categories should not reveal brain areas containing material-representing neurons. Therefore, we use two different approaches that can deal with this problem: a multi-voxel pattern analysis approach and an adaptation paradigm.

In contrast to earlier studies, we used photographs of material surfaces as our stimuli, rather than materials rendered on the surface of virtual 3D-shapes. Typically, rendered textures are much more homogeneous within and across samples than their real-world counterparts. In our image database, we strove for a large variety of different samples that could be easily identified by human observers.

We investigated whether parahippocampal and visual cortex are involved in material perception with two separate experiments. In the first experiment, we employed a multi-voxel pattern analysis approach, in which the pattern of voxel activation is used as the basis for predicting the material category that was seen by the observer. This experiment is an extension of the Hiramatsu et al. study. It was aimed at generalizing their finding of V1 coding to close-up photographs of materials and searching for additional brain regions involved in material perception. Hiramatsu et al. used a limited number of samples per material category. Thus, their classification results were partly based on classification of brain activation patterns to repeated (i.e., identical) images and not on patterns elicited by different images from the same material category. This may have biased their results toward early visual areas, which are known to be retinotopically organized and to be responsive to simple stimulus characteristics such as contrast and orientation. In the second experiment, we employed an adaptation paradigm in which observers adapt to particular material categories. Since an adaptation paradigm has been used previously to demonstrate texture-sensitivity in parahippocampal regions (Cant et al., 2009), we expected that this paradigm would be appropriate for finding material adaptation in the parahippocampal gyrus. Throughout the experiments, we are interested in finding additional areas responsive to material categories.

## Experiment 1: material categorization

### Introduction

A multi-voxel pattern classification approach is suitable for revealing a brain region's responsiveness to manipulations that cannot be revealed with standard contrast approaches. In such a classification approach, the pattern of voxel activations subsequent to an event such as the presentation of a certain stimulus is used to predict the nature of this stimulus. Previous pattern analysis paradigms have, amongst others, revealed neural assemblies responding to orientation (Haynes and Rees, 2005; Kamitani and Tong, 2005) and to different visual scenes (Walther et al., 2009).

We designed an experiment in which observers were presented with stimuli once every 5 s. We asked participants to categorize the materials as either wood, stone, metal, or fabric. We chose to do this explicit classification task to ensure that all neuronal circuits for material processing, whether conscious or not, contribute to the brain activation pattern. Close-up photographs of materials are nearly in exact visual correspondence with the original natural objects, avoiding any complications that arise with computer renderings of such objects. During the experiments, we showed each photograph once, in order to prevent over-fitting of the classification algorithm. We wanted to ensure that the classifiers would generalize to novel samples from the same material categories. We performed full brain scans, in order to potentially find all brain regions involved in material categorization.

### Methods

#### Stimulus presentation

Stimuli were projected with an XGA-Projector (Epson, Model 7250, resolution: 1024 × 768) onto a back projection screen (460 × 350 mm) behind the scanner, which could be seen by means of a double mirror attached to the head coil (visual field 18° horizontal and 16° vertical, rectangular aperture).

Stimulus delivery and response registration were handled by Presentation software (Version 12.2; Neurobehavioral Systems TM, Albany, CA, USA)

#### Stimuli

**Figure 1:**
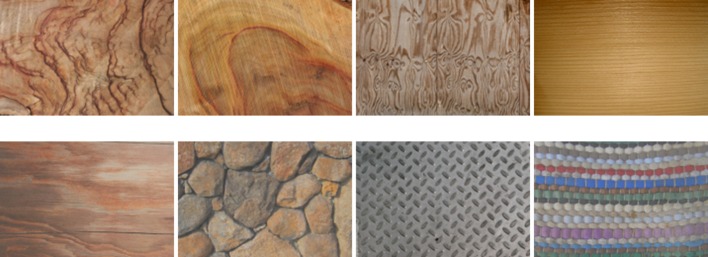
**Example stimuli**. The first row shows wood samples. The second row shows one sample from each category (wood, stone, metal, fabric). The first row is an example of a possible presentation sequence in a material adaptation block, while the second row is an example of a possible presentation sequence in a no-adaptation block (in Experiment 2 below). See http://www.allpsych.uni-giessen.de/MID/ for our stimuli.

Pictures of material stimuli (wood, stone, metal and fabric) were taken with a Nikon D70 camera (Nikon, Tokyo, Japan), under natural conditions (Wiebel et al., 2013). In addition, pictures were obtained from the internet (http://www.textureking.com/, http://textures.forrest.cz/). The stimuli were normalized for mean luminance and luminance contrast (i.e., the standard deviation of the luminance distribution), based on the luminance output of the projector. Only pictures which were categorized correctly by four out of four observers, in a preparatory study, were included in this experiment. See Figure 1 for example stimuli.

#### Participants

Four male participants, all right-handed as assessed by the Edinburgh Handedness Inventory (Oldfield, 1971), enrolled in this experiment. Ages ranged from 20 to 26 years. Vision was normal or corrected to normal. All participants gave their written informed consent. The experiment was approved by the local ethics committee.

#### fMRI-procedure

Each stimulus was presented only once, to ensure correct generalization to other samples of the same category, eliminating the possibility that brain activation patterns for certain material classes could be based on repeated presentations of the same stimulus. In total, 108 images were shown per category. In order to prevent anticipation of the upcoming material category, images were presented in fully random order. Each image was followed by a display of the response options, consisting of four white squares signifying the four material categories. The material category names were written below each square. The duration of the image and the subsequent response phase each lasted 2.5 s. The participant's task was to decide which material category (s)he saw, and to press the corresponding button. As soon as a button was pressed, the corresponding box turned blue.

#### Scanning parameters

Anatomical and functional scans were acquired with a SIEMENS Symphony 1.5 Tesla MR imaging system with a quantum gradients system. The anatomical scan consisted of 160 T1-weighted sagittal images, measured by means of a MP-RAGE sequence. Slice thickness was 1 mm. A field map scan was acquired to correct for inhomogeneities. The functional scan was conducted using a single shot T2^*^-weighted gradient-echo planar imaging (EPI) sequence, with 25 slices covering the whole brain (slice thickness 5 mm; 1 mm gap; *TA* = 2.4 s; *TR* = 2.5 s; *TE* = 55 ms; flip angle 90°; field of view 192 × 192 mm; matrix size 64 × 64; Voxelsize 3 × 3 × 5 mm.).

#### Preprocessing of the data

DICOM-files were converted to NIFTI-files using MRI-Convert (Version 2.0, Lewis Center for Neuroimaging, Oregon). SPM8 (Statistical Parametric Mapping; Welcome Department of Cognitive Neurology, London, UK) was used to pre-process the data. Pre-processing consisted of inhomogeneity correction, unwarping, realignment, co-registration, and normalization to the MNI-template brain. No smoothing was applied.

#### Data analysis

EPI-sequences were linearly detrended, after which voxel activations (taken 5 s after stimulus presentation, when the BOLD-response reaches its peak) were fed to a linear naïve Bayes classifier (Matlab statistics toolbox) for predicting the observed material categories, using a leave-1-out procedure. This was done both for regions-of-interest, and for the entire brain. As regions-of-interest we selected V1, V2, V4, and the parahippocampal place area. In a pilot experiment, we obtained retinotopic scans in two participants. Delineation of visual areas based on this retinotopy did not improve classification accuracy over delineation based on probabilistic cytoarchitectonic masks. Hence, we used probabilistic cytoarchitectonic masks (Eickhoff et al., 2005) for delineating the visual areas and the posterior parahippocampal place area. For the full brain analysis a stepwise regression analysis was performed, i.e., the entire volume was iteratively searched for the voxels yielding the highest increase in classification accuracy. To speed up the algorithm, we restricted classifications to the voxels showing significant (*p* < 0.05 for at least one contrast) activation differences between at least two materials. We based this on direct *t*-tests between the activation accompanying each of the presented material categories. We also excluded voxels outside the head from analysis. In each iteration, the selected voxel was added to the voxels selected in previous iterations This procedure was repeated until there was no further increase in accuracy. To control for overfitting, the same procedure was repeated with randomly permuted material category labels, and the real data were compared to the permuted data. In cases where real data fell in the top 5% of the accuracy distribution results were deemed significant. For the full brain analysis, we present results from individual observers separately, besides the group results.

### Results

#### ROI analysis

**Figure 2:**
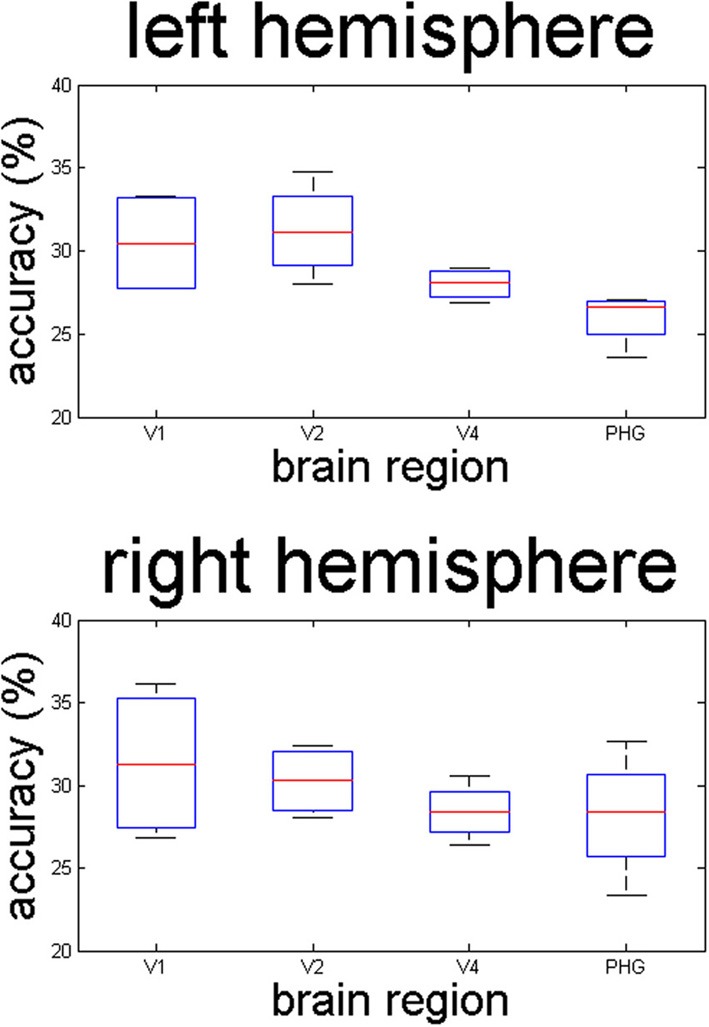
**The linear classifier's prediction accuracies for the presented material categories, based on voxel activations in V1, V2, V4, and the parahippocampal place area**. Results for the left hemisphere are shown on top; the results for the right hemisphere at the bottom. Error bars indicate the standard error of the mean, across subjects. Chance performance is 25% accuracy.

Activation patterns in V1 were the best predictor of the material category observed by the participants. The region-of-interest analyses in the right hemisphere yielded classification accuracies of 31% correct for V1 (*p* = 0.04, one-sided *t*-test), 30% for V2 (*p* = 0.008), 28% for V4 (*p* = 0.014), and 28% for the posterior parahippocampal gyrus (*p* = 0.09), compared to a chance level of 25% - analysis of permuted data indicated that the chance level was indeed 25%. In the left hemisphere, accuracies were 30% for V1 (*p* = 0.02, one-sided), 31% for V2 (*p* = 0.01), 28% for V4 (*p* = 0.004), and 26% for the posterior parahippocampal gyrus (*p* = 0.15). Classification accuracy drops as one moves in an anterior direction through the brain (see Figure 2).

#### Full-brain search analysis

**Figure 3:**
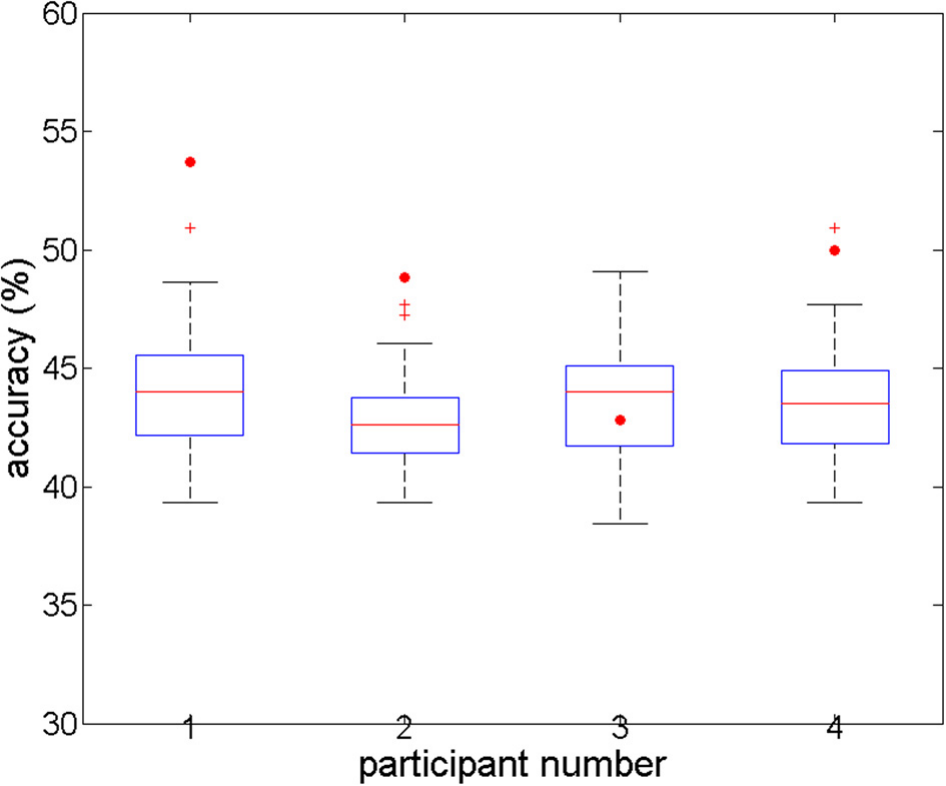
**Overall accuracies in the full-brain search analysis, for four subjects' real and permuted data**. For subjects 1, 2, and 4, prediction accuracy is significant (*p* < 0.05; *n* = 100 permutations). For subject 3, there is no difference between real and permuted accuracies.

For the full-brain search analysis, higher accuracies were obtained (Figure 3). Classification accuracies started around 35% for the first component (voxel) in each participant, and increased to 53.7, 50, 42.82, and 48.84%, with 9, 6, 5, and 10 components, respectively. The corresponding accuracies for the individual permutations reached values between 38 and 51%. For three participants (numbered 1, 2, and 4), the accuracies for the real data were significantly higher than all the corresponding accuracies for the permuted data (*p* < 0.001). For the third participant, the accuracy for his real data was below the average and the median of the accuracies for his permuted data, which was not a significant result. Comparing the accuracies of the four participants to the combined accuracies of their permuted data yielded a highly significant difference at the group level (*t* = −5.0, *p* < 0.001).

**Figure 4:**
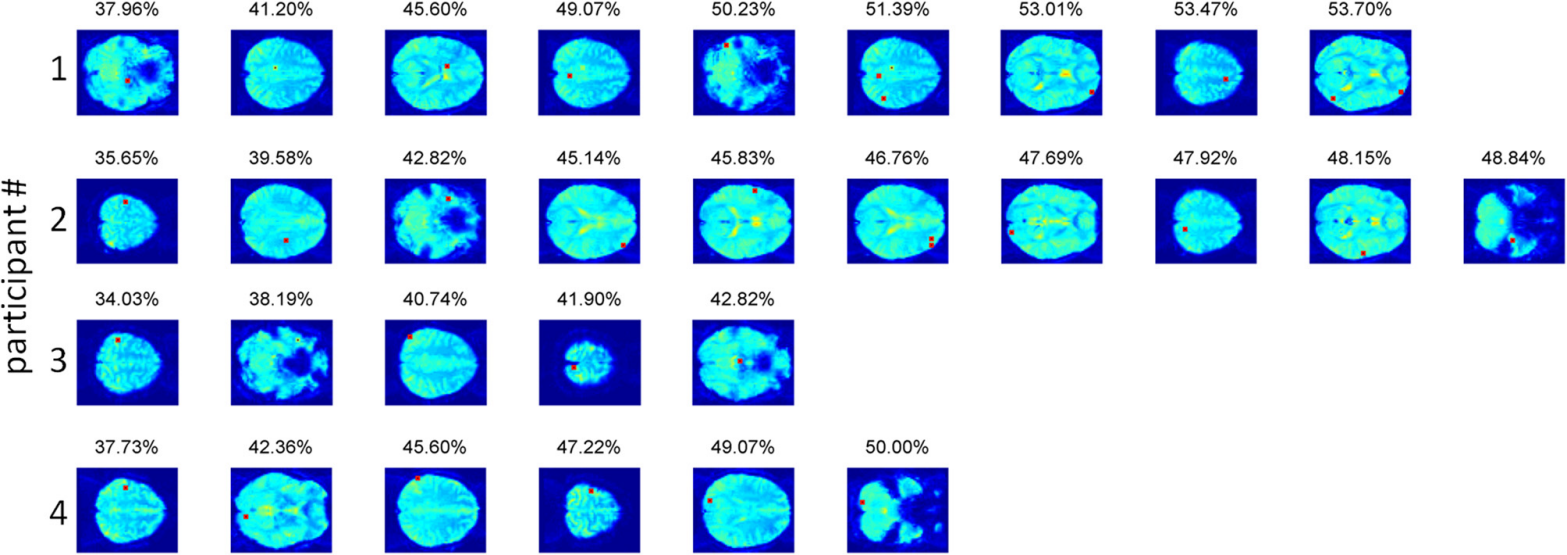
**The voxels contributing to the classification accuracy in the full-brain search approach**. Voxels are displayed in the order of selection by the algorithm, with the obtained accuracy for the combined voxels displayed above the last selected voxel. In participants 2, 3, and 4 the precentral gyrus contains the voxel explaining most of the variance in the material categorization. In participants 2 and 4 early visual cortex participates in the prediction. For participant 2, this voxel (#7) lies in the calcarine sulcus. For participant 4, the voxel (#2) lies in the lingual gyrus. The supramarginal gyrus was present in participants 1 (voxel #6) and 4 (voxel #3).

We should be careful when examining the voxels contributing to the obtained accuracy (Figure 4), as higher than chance accuracies (up till 44% compared to a chance accuracy of 25%) were also obtained for the permuted data, indicating that our procedure fitted noise to some extent. The voxels contributing to the overall accuracy were located throughout the brain in individual participants (see Figure 4). This pattern was anticipated, because neighboring voxels tend to show highly correlated activation, so once a particular voxel has been selected, its neighbors are unlikely to contribute much additional information. Two out of three subjects with significant prediction accuracy had a voxel in early visual cortex contributing to the classification. The same two participants had a contributing voxel in the left precentral gyrus, as one would expect given that motor responses corresponded to particular material categories. In addition, two participants had a voxel in the supramarginal gyrus contributing to the classification. Other than these findings, little consistency could be discerned between the contributing voxels in the different observers.

### Discussion

The classification in regions-of-interest showed that V1 activation was the best predictor for the observed material categories. This finding corroborates (Hiramatsu et al., 2011) finding that V1 was the best predictor for material categories. Importantly, we found V1-involvement even though we presented each material sample only once, thereby eliminating the possibility that the classifier's performance was based on particular samples that were repeated. Hiramatsu et al. used only three samples per material category, so their results could have been due to the repetition of a small number of samples, for example based on simple retinotopic information. Since we presented each sample only once, this complication was avoided. Hiramatsu et al. projected synthesized materials on cylinder-like shapes. Here, we extend their findings to close-up photographs of materials occurring in the environment, usually in the absence of object outline shapes. This excludes the possibility that aspects, like the frequency difference between the material texture (which should be relatively high) and the object outline (with a relatively low frequency), play a major role in V1 pattern activation. It also excludes the possibility that peculiarities in the synthesized stimuli used by Hiramatsu et al. were solely responsible for their results. Another important difference from Hiramatsu et al.'s findings is that we normalized our pictures for overall luminance contrast. This excludes the possibility that overall contrast is responsible for V1 pattern activation, a very real possibility as V1 is known to respond to contrast. High-frequency variations in local contrast could still play a role in both Hiramatsu et al.'s and our own findings, though. We will come back to the issue of spatial frequency information in experiment 3 and in the General Discussion.

The full-brain search confirmed that voxels in V1 contributed to good classification accuracy. This analysis also highlighted voxels in the precentral gyrus, likely related to the different button presses to the different material categories. Another region highlighted in the full-brain search is the supramarginal gyrus. The supramarginal gyrus shows more activation during roughness than during beauty judgments (Jacobs et al., 2012), suggesting it is involved in processing textured aspects of stimuli. However, the supramarginal gyrus appears to be more strongly involved in deriving shape from texture than in discriminating textures (Gulyas et al., 1998), and it also does not seem to be required for tactile material identification (Platz, 1996). Hence, although the supramarginal gyrus appears to process some texture-related aspects of stimuli, it does seem to process them for assessing shape, rather than for discriminating textures. If this is true, then the pattern of activation in the supramarginal gyrus that distinguishes between materials may be automatic and not required for successful performance of the task. Besides these findings, there was little consistency between observers in the location of these voxels. Like Hiramatsu et al., we found that regions anterior to V1 (in our case V2, V4, the parahippocampal place area) contributed less than V1 to material classification. As we mentioned in our introduction, one would expect that the brain codes for materials, not just based on low-level features such as contrast, orientation, spatial frequency, or color, but in a more abstract, categorical manner. There was some evidence for material categorization outside the visual areas, in particular in the supramarginal gyrus, but the evidence was not very strong. Therefore, we opted for a different approach. In a second experiment, we employed an adaptation paradigm to reveal additional brain areas involved in coding categorical differences between materials.

## Experiment 2: material adaptation

### Introduction

Adaptation paradigms have been successfully applied in fMRI research to differentiate different populations of neurons that reside in the same anatomical location. Essentially, a neuron that prefers a particular stimulus feature will initially show strong responses during presentation of the feature, but its activity will diminish during subsequent observations of the same feature. Hence, by comparing conditions in which the preferred stimulus aspect changes continuously to conditions in which the preferred aspect does not change, one can reveal brain areas involved. Adaptation paradigms have been used to demonstrate which brain areas respond to first and second order motion (Ashida et al., 2007), orientation (Fang et al., 2005), and facial identity and expression (Winston et al., 2004), amongst others. Most interesting to our experiment is Cant and Goodale's (Cant et al., 2009) finding that the parahippocampal place area adapts more to texture than to shape, suggesting that this area is specialized in processing texture information. As texture information must be important for categorizing materials, the adaptation paradigm promises to reveal areas outside the visual cortex that are involved in processing materials. Different outcomes do occur for pattern analysis (as used in Experiment 1) and adaptation approaches (Epstein and Morgan, 2011), so the adaptation paradigm is a viable candidate for finding additional regions responding to material categories.

Since we are interested in the brain's response to materials, we used blocks of trials in which the same material (for example, wood, stone, metal, or fabric) was presented repeatedly, and other blocks, in which materials were presented in alternating fashion. To minimize effects of expectancy (the type of material is predictable in the material adaptation blocks), the order of presentation for alternating materials was fixed such that wood preceded stone, stone preceded metal, and metal preceded fabric, after which the cycle repreated. By comparing the brain's activation in the alternating condition to the brain's activation in the condition with the same material, we can reveal the brain areas that code for material categories or their associated properties.

### Methods

#### Participants

Eight male and 14 female participants—with normal or corrected to normal vision—enrolled in our experiment. Their ages ranged from 19 to 30. One male participant was left-handed, as assessed with the Edinburgh Handedness Inventory (Oldfield, 1971). All participants gave their written informed consent. Methods and procedures were approved by the local ethics committee.

#### Stimuli and stimulus presentation

The stimuli, stimulus presentation, and scanning parameters were identical to those in Experiment 1.

#### fMRI-design

Three main conditions were employed. In baseline adaptation blocks the same material image was presented repeatedly. In material adaptation blocks, different images of the same material category (wood, stone, metal, or fabric) were presented. In no adaptation blocks, images of different material categories alternated.

#### fMRI-procedure

**Figure 5:**
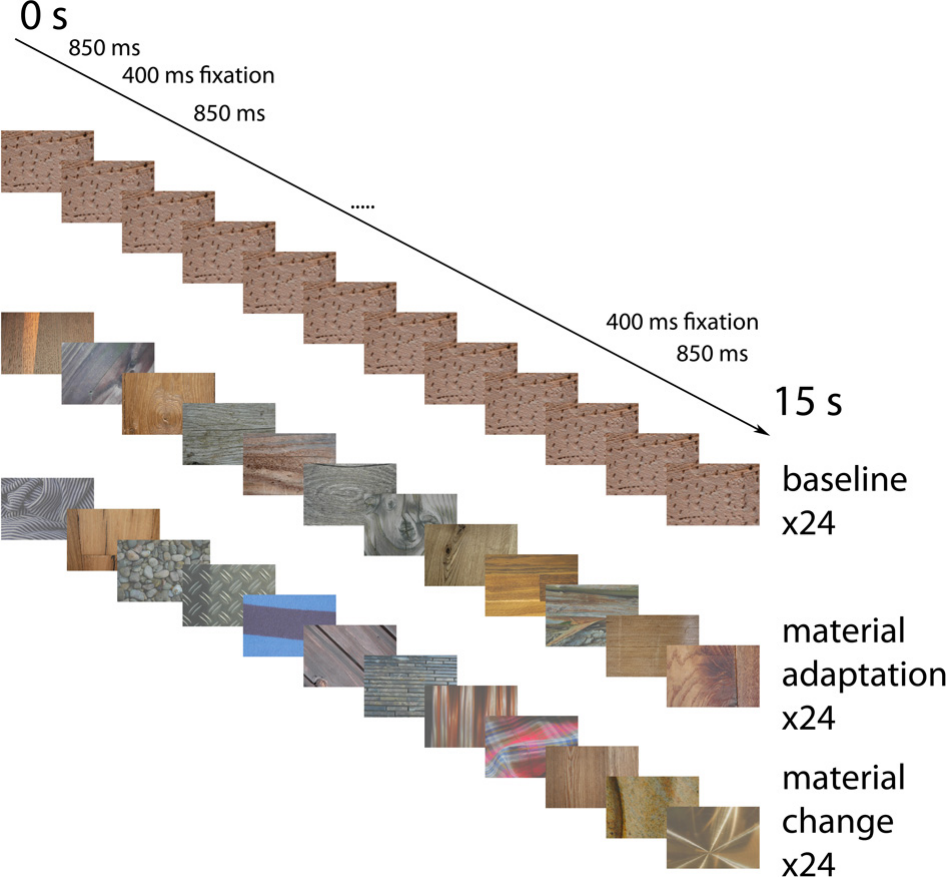
**Example event sequences for the different conditions**. Possible event sequences are shown for the identical images (top), the different material surfaces from the same category (middle) and the materials from different categories (bottom). Not displayed are the fixation screens occurring for 400 ms in-between the stimuli, and the 15 s fixation blocks separating the blocks. Block order was pseudo-randomized in such a way that each type of block was equally likely to follow each other type of block.

Material images were presented in blocks lasting 15 s each. Each block was followed by a fixation period lasting 15 s, during which a uniform gray screen was presented. There were 24 material adaptation blocks, 24 blocks with identical stimuli (each block had a different stimulus), and 24 blocks with alternating material categories. Each material category occurred equally often in each of these conditions. In each block, 12 images were presented. See Figure 5 for an overview of the events. Each image was presented for 850 ms, followed by an interstimulus interval of 400 ms, during which a uniform gray screen was presented. These times were chosen to match the timing of Cant and Goodale (Cant et al., 2009) as closely as possible, while remaining synchronized with the scanner pulses. In the baseline condition, the exact same image was presented 12 times in a row. In the material adaptation condition, different images from the same material category were presented. In the no-adaptation condition, images from different materials succeeded each other. To equate the anticipation of the upcoming material between the different conditions, we used a fixed order of material categories (wood-stone-metal-fabric) in the no-adaptation condition. Participants were informed about this order before the start of the experiment.

To assess whether or not participants kept fixation and paid attention to the screen, they had to indicate by button press when a centrally presented O transformed into a C, as well as the side of the opening (left or right). The O was present during both the material picture blocks and the fixation blocks (uniform gray screen) between. This O-C transformation happened 4 times in each block.

#### Data analysis

DICOM-files were converted to NIFTI-files using MRIConvert (Version 2.0, Lewis Center for Neuroimaging, Oregon). SPM8 (Statistical Parametric Mapping; Welcome Department of Cognitive Neurology, London, UK) was used for pre-processing of the data. Pre-processing consisted of unwarping, realignment, co-registration, and normalization to the MNI-template brain. Nine millimeter FWHM spatial smoothing was applied.

At the group level, a contrast between the no-adaptation and the material-adaptation condition was specified. The adaptation during baseline was used only to verify that activation in the other conditions was higher than this baseline, and is not used in plots. Analyses for such adaptation effects were performed in the posterior parahippocampal gyrus, (Juelich atlas, labeled with SPM anatomy toolbox), with the *p*-value set at a threshold of 0.05. In addition, adaptation effects in the rest of the brain were examined at an uncorrected *p*-value of 0.001. Plots showing the adaptation effect over the course of a block were generated by averaging activation over the no-adaptation blocks, and subtracting this average activation from the average activation during the material adaptation blocks.

### Results

**Figure 6:**
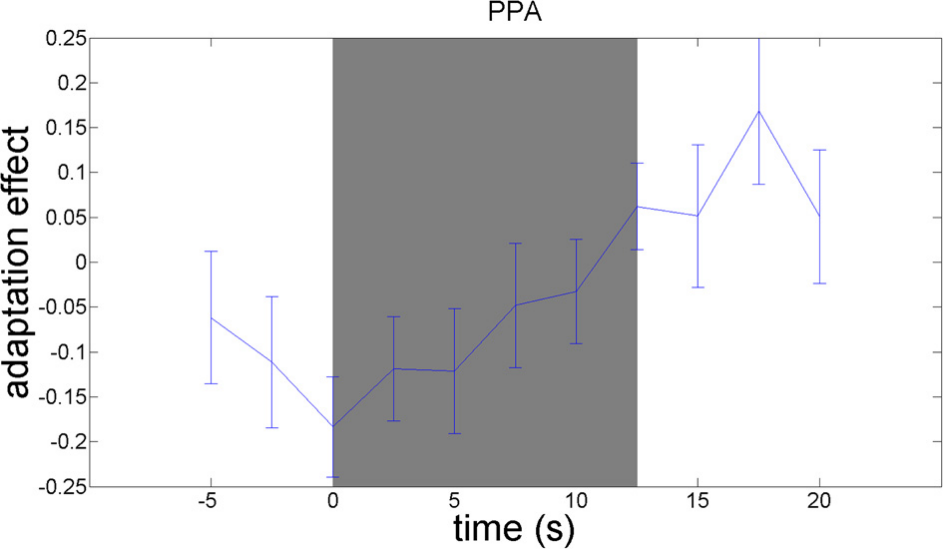
**Timecourses of the contrast between the no-adaptation conditions and the material adaptation conditions, in the parahippocampal gyrus**. The difference in activation between the no-adaptation blocks and the material adaptation blocks is plotted. Blocks start at 0 s and run until 12.5 s. The peak of the BOLD-response is delayed 5–6 s relative to these times. A material image was shown every 1.25 s.

We used an adaptation paradigm to examine material-specific adaptation to wood, stone, metal, and fabric. The contrast between the no-adaptation conditions (with alternating materials) and the material adaptation conditions (with different samples from the same material category) should reveal material-specific adaptation effects. This contrast yielded a significant adaptation effect in the right parahippocampal gyrus, as hypothesized [*t*_(21)_ = 3.13; *p* = 0.005]. A plot of the adaptation over time (Figure 6) reveals that material-specific adaptation accumulates from the beginning of the block until 5 s after termination of the block, when the BOLD-response to the last stimulus in the block is expected to peak.

Examination of adaptation effects in other brain regions, at a statistical threshold of *p* = 0.01, uncorrected for multiple comparisons, suggested adaptation effects in the pre- and postcentral gyrus, the supramarginal gyrus, the cerebellum, and in inferior parietal cortex (see Supplementary Materials).

### Discussion

Neuronal circuits in the parahippocampal gyrus adapted to repeated presentations of the same material category, as hypothesized. This finding confirms that the parahippocampal gyrus performs processes important for material categorization. An obvious candidate is texture processing, as this area has already been implicated for that (Cant and Goodale, 2007; Arnott et al., 2008; Cant et al., 2009), but it may be involved in other aspects of material processing, too. Other areas that showed a material adaptation effect (at *p* = 0.001, uncorrected for multiple comparisons) were the right supramarginal gyrus, the inferior parietal cortex, the right cerebellum, and the pre- and postcentral gyrus (see supplemental information). These results should be considered tentative, considering our lenient threshold. The postcentral gyrus is known as primary somatosensory cortex, and it has been mentioned in connection with the tactile processing of texture information (Lederman et al., 2001). We are not the first to report that somatosensory cortex is responsive to nontactile stimulation. An earlier report demonstrated the response of somatosensory cortex to the sight and sound of someone else being touched (Keysers et al., 2010). Since our stimuli were material surfaces, its activation is not surprising, since observers may imagine touching the surfaces. Visual imagery is known to activate brain areas involved in visual perception (Kosslyn, 1996), so haptic imagery may be expected to activate brain areas involved in haptic perception, such as primary somatosensory cortex. If this reasoning is correct, this would be a first case of fMRI-adaptation for imagined content. One may more cautiously speak of “shared circuits” (Keysers, 2011), activated by both visual and tactile perception of materials. The task at hand appears to determine which sensory modality dominates perception (Lederman et al., 1986). For material perception tactile perception may be primary, and a translation of visual input to a haptic code may be necessary to determine what material is perceived. Involvement of the primary somatosensory area in visual material perception mayimply direct visual-tactile mappings. This idea would refute the classical idea of high-level associative cortex integrating information from different sensory modalities, suggesting instead that direct mappings between different sensory modalities might lead to an integrated experience of the outside world. Our finding of the supramarginal gyrus' involvement in both the categorization experiment and the present experiment may indicate that it is a relay station between the visual and tactile areas. It could also be a supramodal area integrating information from different senses.

To conclude, we found that circuits in the parahippocampal gyrus adapted to visually presented material categories. This finding fits well with earlier demonstrations of the area adapting to texture information. Besides the parahippocampal gyrus, we find the supramarginal gyrus, the primary somatosensory cortex, the motor cortex and the cerebellum may be involved in visual material perception.

## Experiment 3: the effects of high-frequency information

**Figure 7:**
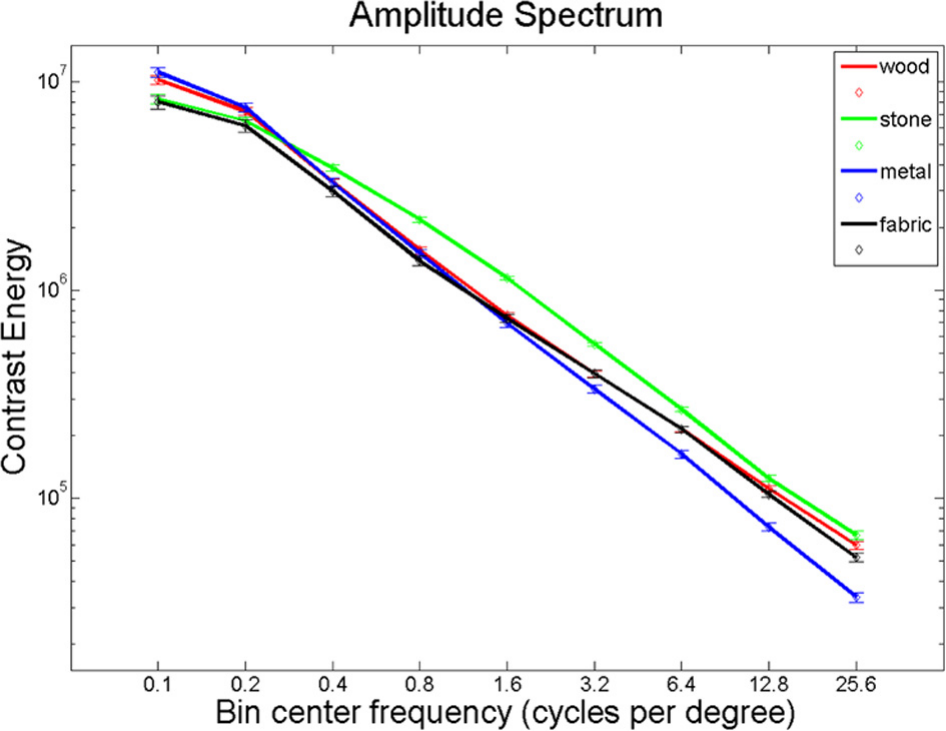
**Energy in the spatial frequency bands for the material images, per material category**.

Giesel and Zaidi (2013) found that manipulations of spatial frequency result in alterations in perceived material properties. We hypothesized that material perception relies largely on high-frequency texture information. Using Fourier analysis, we computed the energy in eleven spatial frequency bands for each of our images and found significantly different distributions between the material categories in all spatial frequency bands (Figure 7; Kruskal-Wallis tests, all *p* < 0.001, except for the range 0.3–0.5 cycles per degree, where *p* = 0.024). Given these differences, it seems plausible that the brain uses this information to distinguish between material categories. If this is the case, one would expect overlap between areas that distinguish materials and areas that respond to spatial frequency information, particularly the higher spatial frequencies that define textures.

To test whether distinctions between material categories are based on differences in spatial frequencies, we designed an experiment to examine the extent to which regions in the previous experiments overlap with regions coding high-frequency texture information. Our stimulus set contained images of various natural objects, gathered from the Internet. In the first condition, smoothed versions of the pictures (i.e., spatially low-pass filtered) were presented twice in succession. In the second condition, the smoothed image was presented, and it was followed by the original image. We intended to increase the salience of each texture, while leaving outline information relatively constant across conditions. Comparing both conditions should reveal texture regions along the collateral sulcus, in the medial visual cortex, as explained in the General Introduction.

### Methods

#### Subjects

The subjects participating in Experiment 2 performed this task in the same session.

#### Stimuli

**Figure 8:**
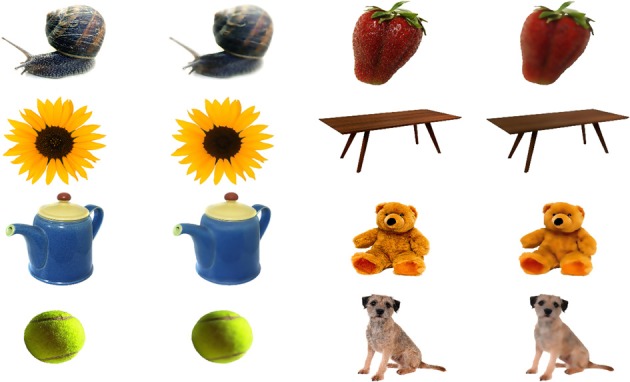
**Examples of original and filtered images used in Experiment 3**. The stimuli were selected from various Internet sources.

**Figure 9:**
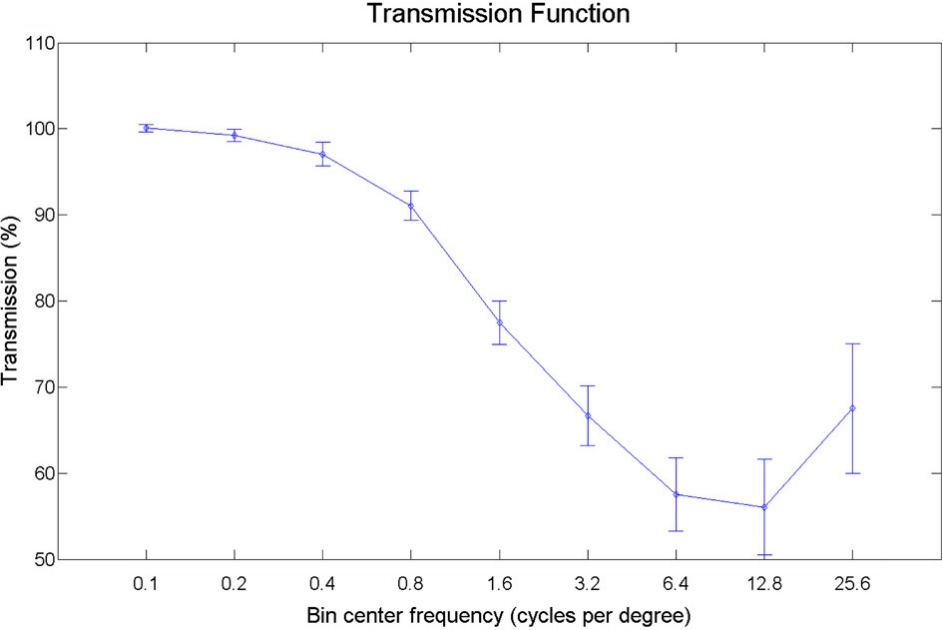
**Transmission plot**. The energy per spatial frequency in the filtered images expressed as a percentage of the energy in the original images, averaged over orientations.

Pictures of animals, fruits, and objects were gathered from the Internet. We selected 60 pictures with substantial detail and texturing. Smoothed versions of the images were obtained by filtering out texture-frequencies while leaving edge information relatively intact, using Tomasi and Manduchi's method (Tomasi and Manduchi, 1998). Examples of the stimuli used are shown in Figure 8. We expressed contrast energy in the filtered images as a percentage of the contrast energy in the original images (Figure 9). As can be seen from Figure 9, the lowest spatial frequencies were left relatively intact, while energy at the higher frequencies was lowered.

#### Procedure

Pictures were presented twice in succession, separated by a gap of 250 ms. Each image presentation lasted 1000 ms. Trials were separated by a gap of 2000 ms, +500 ms multiplied by a random whole number ranging from 1 to 6, inclusive. The smoothed version of the picture was always the first of the two images in each trial. In the baseline condition, the first and second picture presentations were identical. In the intact texture condition, the second occurrence of the picture comprised the original picture, with the original, unfiltered, texture. We expected this procedure to induce outline adaptation in both conditions. In the baseline condition, spatial frequency information in the range of texture information was minimized. In this texture frequency condition, texture would not be adapted and be more salient to perception and hopefully to neurons encoding material information.

#### Equipment

Scanner and projector were identical to those used in Experiments 1 and 2.

### Results

**Figure 10:**
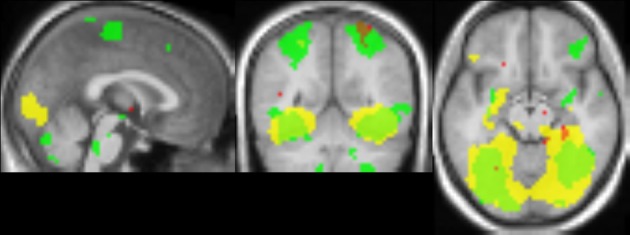
**Comparison of adaptation contrasts to the contrast in activation between perception of intact and smoothed images**. The contrast between material adaptation (same material, different image) and baseline adaptation (same image) is shown in green. The contrast between no adaptation (different materials) and material adaptation is shown in red. The contrast between perception of intact and smoothed images is shown in yellow. A contrast threshold of *t* = 3.00 was used at MNI-coordinates *x* = 0, *y* = −55, *z* = −13. There is a striking overlap between the contrast between material adaptation and baseline adaptation, and the contrast between intact and smoothed images.

The comparison between blocks with objects with intact texture vs. blocks with smooth-textured objects (see Figure 10) yielded activation that overlapped well with activation found in the comparison between material adaptation (different samples of the same material) and baseline adaptation (identical stimulus) obtained in Experiment 2. The overlap with the material adaptation contrast (different materials vs. different images of the same material) is much lower. Also when lowering the threshold for this contrast, the overlap remained low, with the center of mass lying much more anteriorly (not shown).

### Discussion

As expected, spatial frequency information in the range of texture information activated areas in the medial visual stream, where texture is thought to be processed.

Our hypothesis that spatial frequencies in the range of texture information are important for distinguishing between materials was falsified. Rather, the overlap in activation during the perception of intact vs. smoothed objects and material adaptation vs. baseline adaptation conditions suggests that spatial frequencies in the range of texture information are more important for distinguishing between different exemplars within the same category than for distinguishing between different material categories.

## General discussion

In three experiments we examined the neuroanatomical basis for material perception, with a focus on the perception of material categories. We searched for the involvement of visual areas and the parahippocampal gyrus and for signs of the involvement of other brain areas.

Our main findings were that V1 contains information to distinguish between different material categories and that activation of the parahippocampal gyrus adapts to repeated presentation of material categories. In addition, both approaches yielded some evidence for involvement of the supramarginal gyrus in material categorization, and the adaptation experiments pointed to the postcentral gyrus and the cerebellum as areas that may also be involved in material categorization. Given our lenient thresholds for these additional areas, these results necessitate confirmation. However, the supramarginal gyrus is of interest since it was not only active in two out of three participants for our material categorization experiment and in our adaptation experiment, but it also appeared in comparisons of roughness and beauty judgments for visually presented textures (Jacobs et al., 2012). Therefore, it may play a general role in the analysis of textures or material properties.

The results of Experiment 3 indicated that spatial frequency appears to be the basis for distinguishing between different material samples, rather than for distinguishing between different material categories. This conclusion is based on a striking anatomical overlap of activations, and should be verified in behavioral experiments that directly manipulate spatial frequency content.

Although both multivoxel pattern analysis and fMRI-adaptation aim at finding sub-voxelsize activation patterns, we obtained different results using these two approaches, even when using the same stimuli. Our results suggest that the adaptation paradigm is more sensitive to high-level representations. The information represented in the parahippocampal gyrus may be of an abstract nature, reflecting the material categories we used, or at least reflects higher-order statistical features that are highly characteristic of the material categories. We excluded average luminance, contrast, and spatial frequency information as critical features that lead to differential material adaptation in the brain, but many other features could play a role. Multi-voxel pattern analysis seems to be more sensitive to adaptation in low-level features represented in early visual areas, such as local contrast, spatial frequency, and orientation. Such features may, alone or in combination, distinguish between different material categories, but it seems likely that the higher-order representations captured by the adaptation approach are more directly involved. That being said, we should not forget that other differences between our experiments may explain the different findings. For example, during our categorization task the observers were attending to the material pictures, while during our adaptation task, observers were attending Landolt-Cs superimposed on the material pictures. Weigelt et al. (2012) found stronger adaptation effects when observers attended to the relevant dimension, so manipulations of attention might have altered the outcomes of our experiments. Another difference is that our adaptation experiments demanded strong fixation, while during the categorization experiment, central fixation was ordered but not required for successful task performance. Since Hiramatsu et al. obtained very similar results to our categorization experiment (with a fixation task that resembled the fixation task used in our adaptation experiment), we consider it unlikely that differences in fixation played a significant role.

## Conclusion

Both early visual cortex and the parahippocampal place area appear to discriminate between material categories. We demonstrated V1 involvement even under circumstances where a pattern classifier could not rely on the repetition of identical material samples, as in Hiramatsu et al. (2011). Since the classifier results could reflect low-level featural differences between the material categories, without a more abstract distinction between material categories, we sought additional sites of material processing through the use of an adaptation paradigm. This approach revealed involvement of the parahippocampal gyrus, an area which has been implicated in texture perception. The parahippocampal gyrus may contain abstract representations of material categories, or it may code features that happen to be associated with the different material categories employed in our experiment. Regardless, the information contained in the parahippocampal gyrus is likely to be of a higher level than the information coded by V1. Based on anatomical overlap, spatial frequency information in the range of typical frequencies found in textures seems to be more important for distinguishing between samples within material categories, rather than for distinguishing between categories.

Full-brain analysis suggests that the primary somatosensory cortex and the supramarginal gyrus are also involved in visual material perception. However, these results have to be confirmed in future. The discrepancies between Experiment 1 and Experiment 2 indicate fundamental differences in the two approaches to find sub-voxelsize activations in the brain, even when the same set of stimuli is used in both paradigms. While the pattern-classification approach implicated early visual areas in the material coding process, the adaptation paradigm implicated mainly the parahippocampal gyrus. This suggests that the adaptation approach is more sensitive to higher order aspects of stimulus processing, although other differences in our experiments may also be the reason for the differences. Further research into the reasons for such discrepancies is needed.

### Conflict of interest statement

The authors declare that the research was conducted in the absence of any commercial or financial relationships that could be construed as a potential conflict of interest.

## Supplementary material

Figure S1 **Timecourses of the contrast between the no-adaptation conditions and the material adaptation conditions, in inferior parietal cortex**.

Figure S2 **Timecourses of the contrast between th2e no-adaptation conditions and the material adaptation conditions, in the right supramarginal gyrus**.

Figure S3 **Timecourses of the contrast between the no-adaptation conditions and the material adaptation conditions, in the right cerebellum**.

Figure S4 **Timecourses of the contrast between the no-adaptation conditions and the material adaptation conditions, in the left postcentral gyrus**.

Figure S5 **Timecourses of the contrast between the no-adaptation conditions and the material adaptation conditions, in the left precentral gyrus**.

Figure S6 **Timecourses of the contrast between the no-adaptation conditions and the material adaptation conditions, in the right precentral gyrus**.
