# Supplementary material for: The representation of material categories in the brain
Source: Front Psychol. 2014 Mar 12;5:146. doi: 10.3389/fpsyg.2014.00146 (PMC3950415; doi:10.3389/fpsyg.2014.00146)
Supplement: Figure S1 — Timecourses of the contrast between the no-adaptation conditions and the material adaptation conditions, in inferior parietal cortex. [file DataSheet1.DOCX]

# Supplementary information to “Material categories in the brain”

**Figures S1-S 6. Timecourses of the contrast between the no-adaptation conditions and the material adaptation conditions.** The difference in activation between the no-adaptation blocks and the material adaptation blocks is plotted for regions showing significant material adaptation, at the liberal threshold of p = 0.05, uncorrected for multiple comparisons. Blocks run from 0 to 12.5 s (indicated in grey). The BOLD-response is expected to peak 5 to 6 seconds later.


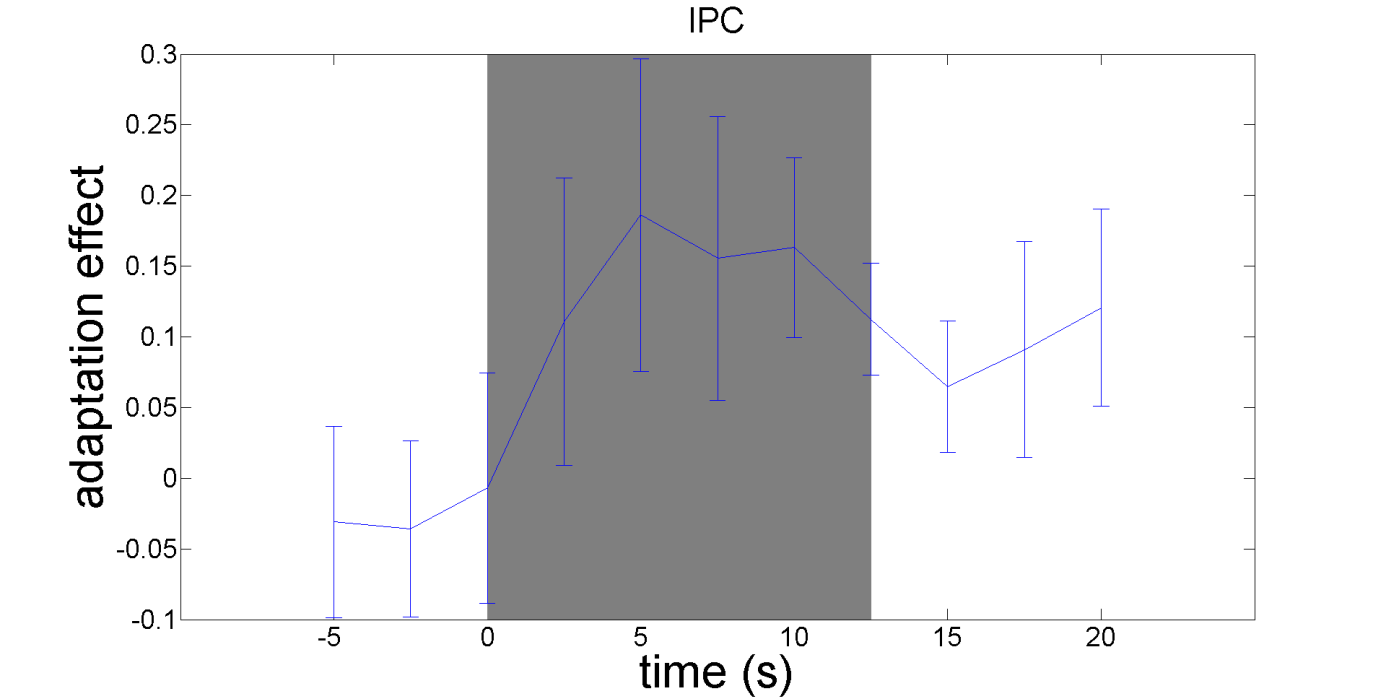


**Figure S1. Timecourses of the contrast between the no-adaptation conditions and the material adaptation conditions, in inferior parietal cortex.**


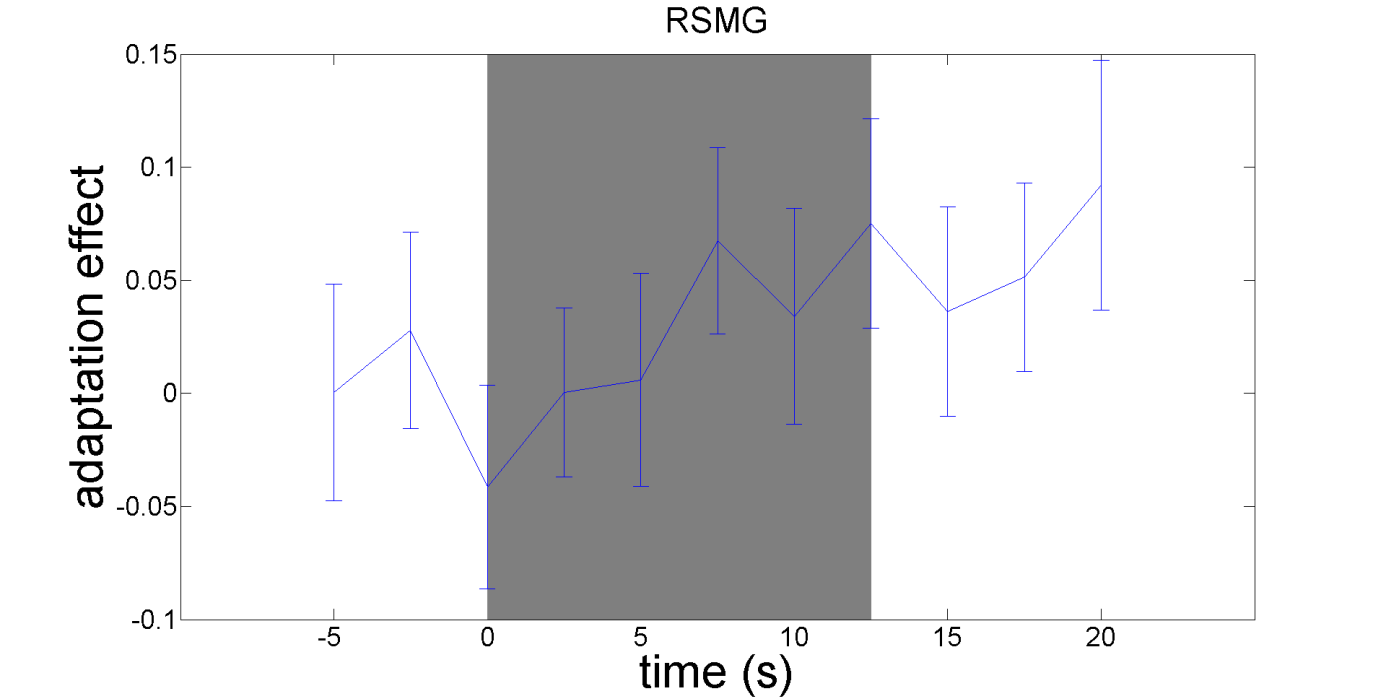


**Figure S2. Timecourses of the contrast between the no-adaptation conditions and the material adaptation conditions, in the right supramarginal gyrus.**


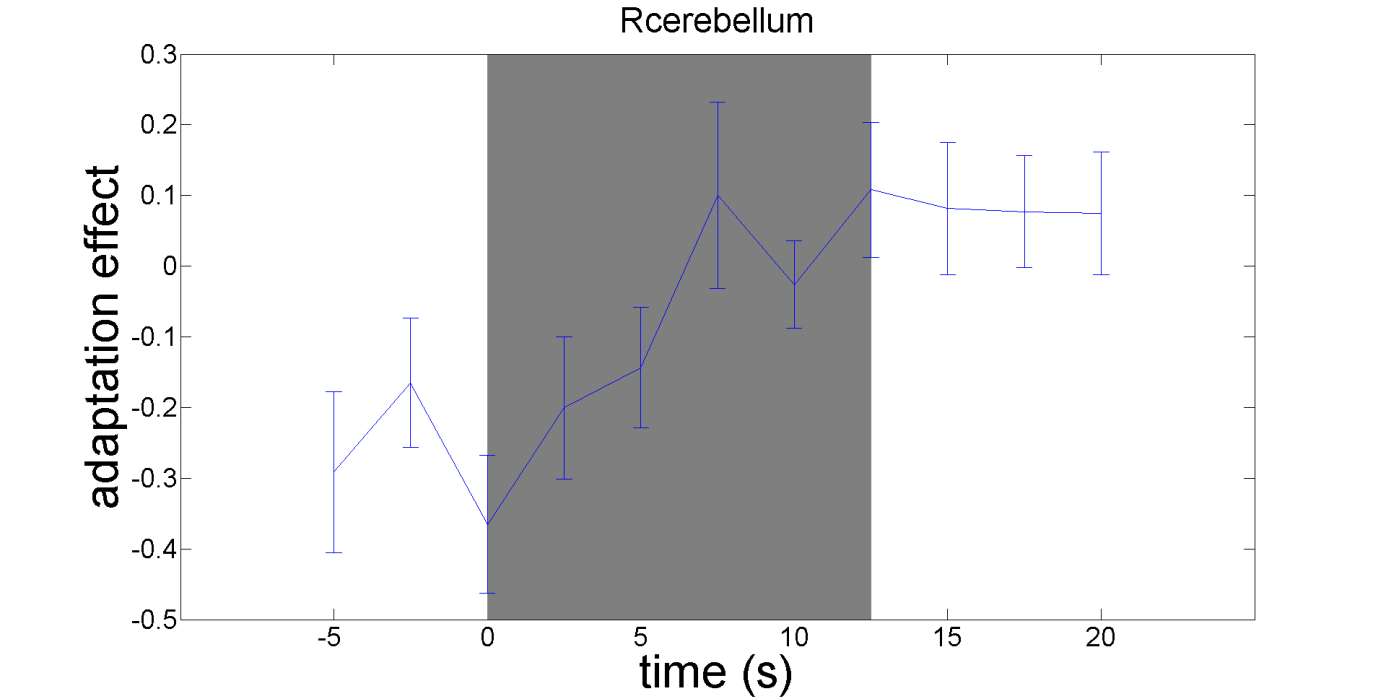


**Figure S3. Timecourses of the contrast between the no-adaptation conditions and the material adaptation conditions, in the right cerebellum.**


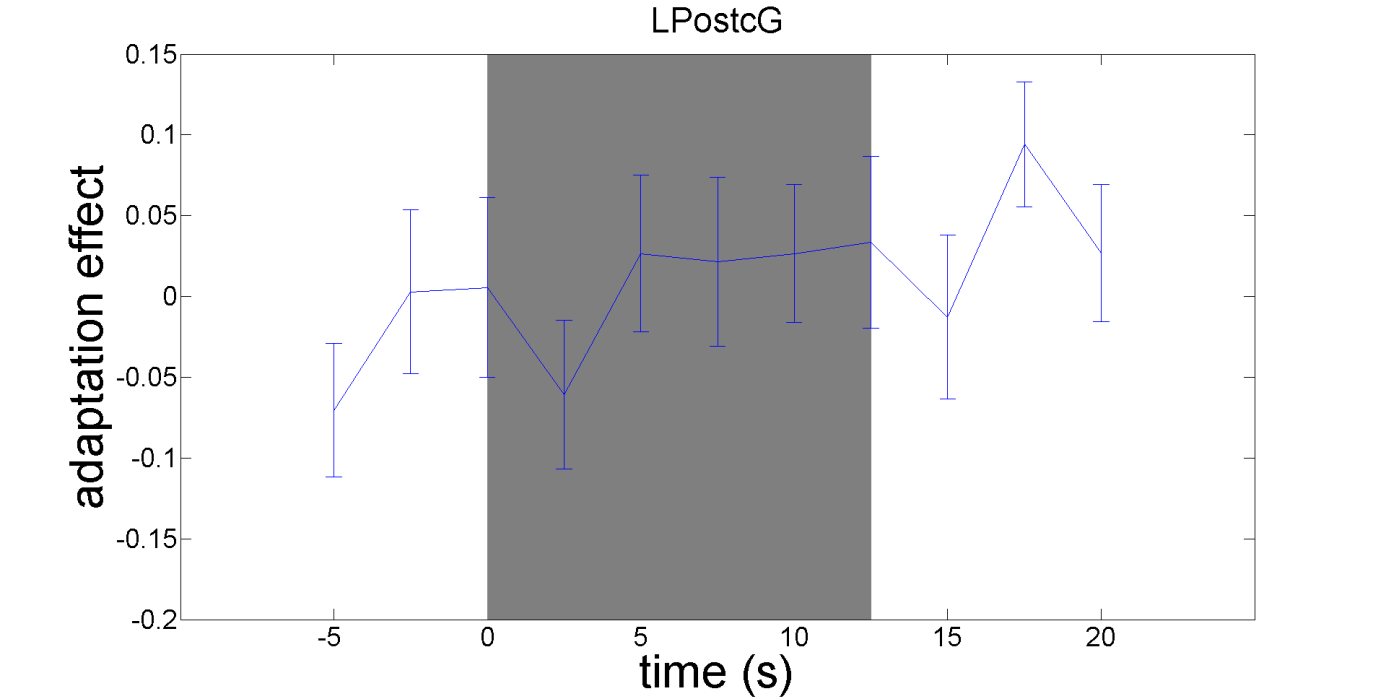


**Figure S4. Timecourses of the contrast between the no-adaptation conditions and the material adaptation conditions, in the left postcentral gyrus.**


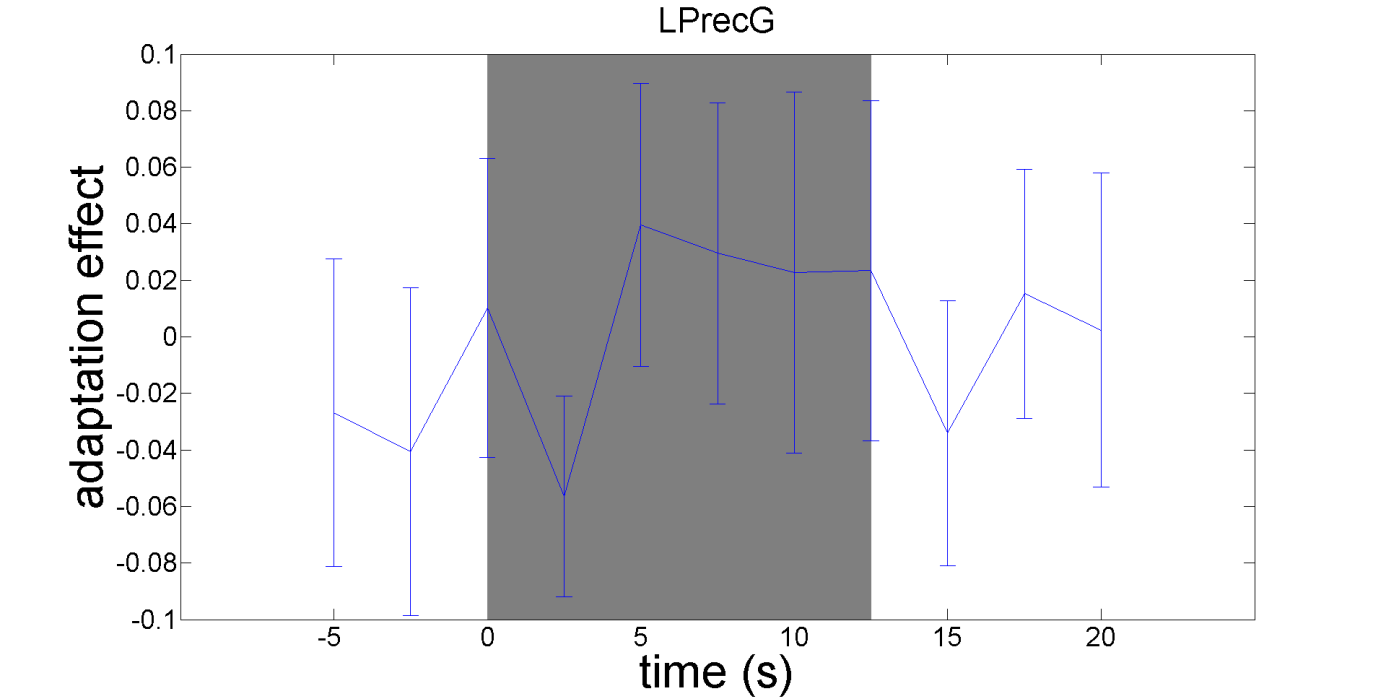


**Figure S5. Timecourses of the contrast between the no-adaptation conditions and the material adaptation conditions, in the left precentral gyrus.**


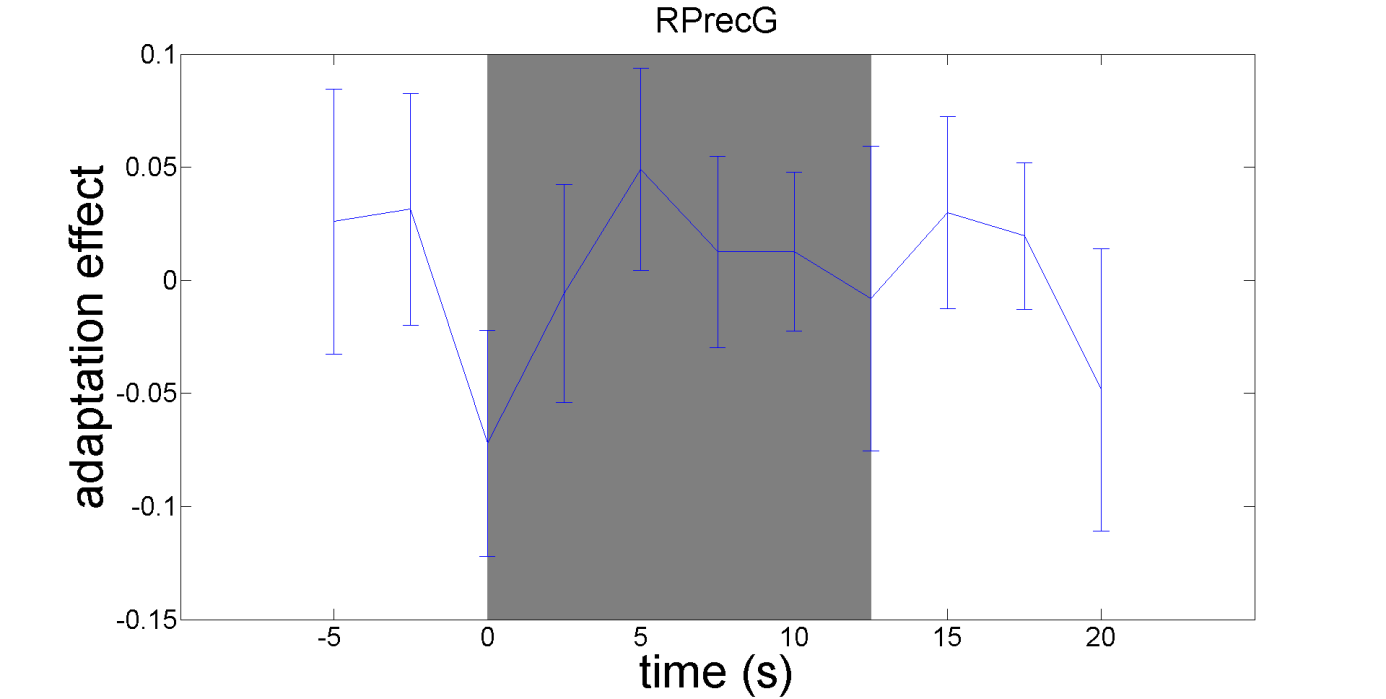


**Figure S6. Timecourses of the contrast between the no-adaptation conditions and the material adaptation conditions, in the right precentral gyrus.**
